# Taurine supplementation enhances anaerobic power in elite speed skaters: A double-blind, randomized, placebo-controlled, crossover study

**DOI:** 10.5114/biolsport.2023.119990

**Published:** 2022-10-14

**Authors:** Yusuf Buzdağlı, Cemre Didem Eyipınar, Furkan Öget, Erdinç Şıktar, Scott C. Forbes, Aslıhan Tekin

**Affiliations:** 1 Department of Coaching Education, Faculty of Sport Sciences, Erzurum Technical University, Erzurum, Turkey; 2 Department of Physical Education and Sport, Faculty of Sport Sciences, Gaziantep University, Gaziantep, Turkey; 3 Department of Physical Education and Sport, Faculty of Sport Sciences, Erzurum Technical University, Erzurum, Turkey; 4 Department of Coaching Education, Faculty of Sport Sciences, Atatürk University, Erzurum, Turkey; 5 Department of Physical Education, Faculty of Education, Brandon University, Brandon, Manitoba, Canada; 6 Department of Physical Education and Sport, Faculty of Sport Sciences, İbrahim Çeçen University, Ağrı, Turkey

**Keywords:** Anaerobic Power, CMJ, Fatigue, Taurine, Wingate

## Abstract

Taurine (2-aminoethanesulfonic acid) is a semi-essential sulphur-containing amino acid abundant in skeletal muscle. Taurine supplementation is popular among athletes and has been purported to enhance exercise performance. This study aimed to investigate the ergogenic effects of taurine supplementation on anaerobic (Wingate; WanT) performance, blood lactate, ratings of perceived exertion (RPE), and countermovement vertical jump (CMJ) in elite athletes. For this study, randomized, double-blind, placebo-controlled crossover designs were used. Thirty young male speed skaters were randomly assigned to either taurine (TAU; single dose of 6 g) or placebo (PLAC; single dose of 6 g) 60 minutes before testing. Following a 72-hour washout, period participants completed the opposite condition. TAU improved peak (Δ% = 13.41, p < 0.001, d = 1.71), mean (Δ% = 3.95, p = 0.002, d = 1.04), and minimum power output (Δ% = 7.89, p = 0.034, d = 0.48) compared to placebo. Further, RPE (Δ% = -10.98, p = 0.002, d = 0.46) was significantly lower following the WanT in the TAU condition compared to placebo. There were no differences between conditions for the countermovement vertical jump. In conclusion, acute TAU supplementation augments anaerobic performance in elite speed skaters.

## INTRODUCTION

Taurine (TAU) is an essential amino acid derived from cysteine metabolism, and this sulphur-containing component is abundant in skeletal muscle [1]. Despite mixed or limited evidence supporting its efficacy, TAU is a popular purported ergogenic aid in athletes [2]. Mechanistically, taurine, generated from cysteine metabolism, plays a vital role in various physiological and metabolic actions [3, 4], including antioxidant pathways, lipid or glucose oxidation, anti-inflammatory modulation, and energy metabolism provision [1]. As such, TAU has been purported to improve exercise performance. Taurine ingestion causes taurine plasma concentrations to rise within 10 minutes and peaks within 60 minutes (0.03 to 0.06 mmoL). Taurine plasma concentrations typically return to baseline after 6.5 hours following ingestion [5].

When performing the high-intensity anaerobic exercise, blood lactate (BLa) and hydrogen ions (H ^+^) concentrations increase, declining the muscle cell pH [6] and lowering its ability to produce adenosine triphosphate (ATP) required for sustained muscle contractions [7]. A 30-seconds (s) Wingate test (WanT), has been widely used due to its ability to assess non-oxidative energy metabolism [8]. Accumulation of H ^+^ during WanT suppresses resynthesis of phosphocreatine (PCr) and phosphofructokinase enzyme activity (which is the rate-limiting enzyme involved in anaerobic glycolysis) and both contributing to muscular fatigue [9]. Specifically, fatigue is defined as a decline in the ability to create power [10]. Furthermore, the countermovement jump (CMJ) test is a practical method for evaluating neuromuscular fatigue (NMF) [11], adaptations associated with training [12], and recovery status [13] making it a handy tool for measuring muscle fatigue/recovery in athletes [14]. Along with jump height, force and power production during the CMJ are directly related to the total jump time. This lower body extremity muscle power output [15]. A decrease in CMJ performance after exercise is considered a helpful tool for monitoring the neuromuscular state [16].

When the experimental [17–19] and meta-analysis [20] studies on taurine are examined, it is seen that there is no consensus on its positive effects in terms of performance. In addition, it is thought that the elite population of our choice and the effect it will have on performance may be the focus of attention of those who do sports at the elite level. In this study, thirty young male speed skaters were subjected to the WanT test after TAU supplementation, and primarily their anaerobic performance was evaluated. There was a reason why especially speed skaters were chosen in the study. Speed skating is divided into two primary categories: short-track and long-track racing [21]. A sports discipline with a higher physiological demand is practiced on a short-track. The biomechanically advantageous crouching position, which is necessary for speed skating performance, must be adopted by skaters [22]. An inclined posture, due to the nature of this sport, results in physiological drawbacks, such as deoxygenation of active muscles [23]. A decrease in tissue pO2 can be detected by changes in muscle oxygenation, which may then be related to performance and fatigue [24]. In these circumstances, glycolysis will serve as the primary energy source for the muscle fibers, potentially aggravating fatigue [23]. It has been stated that taurine has lactate-lowering activity due to its buffering effect [25, 26]. This is probably owing to a potential interaction between taurine and calcium’s function as a buffer in the mitochondria. Taurine can help keep mitochondrial activity intact during exercise and improve the buffering capacities of the cells [27].

In addition, success in short-track skating depends not only on the skater’s excellent riding technique but also, and perhaps more importantly, on the high speed that is created thanks to the power of the lower limbs, which converts into work including anaerobic alterations [28]. Taurine helps release Ca^2 +^ from the sarcoplasmic reticulum, enhancing the sensitivity of force-generating myofilaments in skeletal muscle [4], resulting in an increase in muscular force [3] and better performance results.

Another point that should be mentioned in terms of anaerobic performance is the antioxidant activity of taurine. Studies have indicated taurine’s capacity to serve as an antioxidant, which may create an enhanced cellular environment to handle exercise-induced oxidative stress [29, 30]. Since the speed skater is a high-intensity branch, free radicals are formed. Free radical generation might harm cellular components, following high-intensity exercise [31]. Due to the presence of sulfonic acid, Taurine, which induces the conversion of extremely cytotoxic chemicals like chloride and hypochlorous acid into stable chloramine, and it has been referred to as a powerful antioxidant [32]. The body cannot produce enough taurine under conditions of high intensity exercise, highlighting the need of taurine-based dietary supplements [33]. Hence, using antioxidant supplements, like taurine, after exercise aims to reduce oxidative stress and possibly improve performance [34].

At the same time, TAU is classified as non-essential amino acid and it has an effect on brain metabolism (via regulating neural transmission) [35]. TAU plays the role of an extra synaptic GABAA receptor agonist, which can boost network activity at many brain locations, including the thalamus [36]. Although taurine differs from other amino acid-based supplements in this respect, when we look closely at the literature, there are scarcely any studies examining the effect of taurine on neuromuscular fatigue [33]. Regrettably, research on the effect of fast or practice alternative supplements on sportive performance and brain metabolism is very limited because studies have mostly focused on examining the long-term supplement use effects. Therefore, this study focused on the hypothesis that acute intake of TAU would improve athletic performance without affecting neuromuscular fatigue.

Furthermore, it is unclear whether TAU may be considered ergogenic for elite athletes participating in anaerobic sports (e.g., speed skating) and whether TAU alters post-exercise NMF. Hence, the objectives of this study were: (a) to evaluate the ergogenic effect of TAU on WanT performance in elite athletes and (b) to determine its effects on post-exercise BLa and possible decrease in CMJ. We hypothesize that TAU will improve anaerobic performance (WanT) and BLa concentration without altering exercise NMF.

## MATERIALS AND METHODS

### Participants

Thirty male short-track speed skaters (age: 23.9 ± 2.8 years, body mass: 76.18 ± 2.8 kg, height: 177.54 ± 7.6 cm, BMI: 23.9 ± 3.7 kg/m^2^; training experience:11.4 ± 3.4 years) were a part of Turkey’s Olympic Preparation Centre team volunteered (Table 1). The weekly training program consisted of strength training 2.0 ± 0.1 sessions · wk^−1^ and endurance training 3.0 ± 0.1 sessions · wk^−1^ and was completed by all the participants. Since the sports dietitian controlled the food consumption of all participants, 24-hour dietary recalls were taken in this study. The carbohydrates (g · kg^−1^), protein (g · kg^−1^), fat (g · kg^−1^), and total energy (kcal) values of each food in these records were obtained from the TURKOMP database (http://www.turkomp.gov.tr/database) (TURKOMP is a food composition database that includes nutritional components and energy values of the wide variety of foods produced and consumed in Turkey) [37]. The daily macro-nutrient intakes were calculated in excel sheets for all participants. The average values of macronutrients (carbohydrates (g · kg^−1^), protein (g · kg^−1^), fat (g · kg^−1^), and total energy (kcal)) calculated in Excel are summarized.

**TABLE 1:** Participant Characteristics

Variables	Male athletes (n = 30)	
	mean ± SD	[95% CI]
Age (years)	23.90 ± 2.80	[22.00, 24.00]
Training experience (years)	11.40 ± 3.40	[9.78, 12.99]
Body mass (kg)	76.18 ± 2.80	[75.00, 77.00]
Height (m)	1.77 ± 7.60	[1.74, 1.79]
BMI (kg/m^2^)	23.90 ± 3.70	[21.68, 24.32]
ST frequency (sessions per week)	2.00 ± 0.10	[1.96, 2.04]
ET frequency (sessions per week)	3.00 ± 0.10	[2.96, 3.04]
Total energy intake (kcal)	2900 ± 243	[2813, 2986]
Carbohydrate intake (g·kg^-1^)	4.50 ± 0.10	[3.96, 4.04]
Protein intake (g·kg^-1^)	2.20 ± 0.10	[2.16, 2.24]
Fat intake (g·kg^-1^)	1.00 ± 0.50	[0.82, 1.18]

Abbreviations: The mean and standard deviation [95% CI] are used to represent the data. ST: strength training, ET: endurance training, kcal: kilocalories; g/kg^−1^: grams per kilogram of body mass.

To take part in the study, researchers verbally verified that all of the volunteers met the inclusion/exclusion criteria a) participants were not taking any dietary supplements within the past three months before the start of the study, b) participants did not smoke, c) participants were healthy with no diagnoses of cardiovascular, respiratory, or metabolic diseases which may impair muscle biology, d) had no orthopedic injury that would impact cycling performance and e) had to have previous experience of performing high-intensity exercise. Before signing an informed consent form, all participants were briefed about the procedures and risks. All the experimental testing sessions were carried out at Atatürk University Athlete Performance Measurement Evaluation and Rehabilitation Center. This study was supported by the Research Fund of Ataturk University. Project Number: 10115.

### Study Design

A randomized, double-blind, placebo-controlled, crossover study was used. All testing sessions were completed within a 72-hour time frame to minimize training effects and were finished at the same time of day (within ~0.5 hours) to reduce any biological variability due to circadian rhythm [38]. Each visit was separated by no more than 72 h for each participant, which was deemed sufficient to wash out taurine based on a half-life of between 70 and 100 minutes [18]. Participants were randomized to receive either placebo (PLAC) or TAU supplementation on the first experimental session and the opposite condition on the second experimental session using a computerized randomization software (GraphPad, San Diego, USA; available at: https://www.graphpad.com/quickcalcs/randomize1.cfm).

**FIG. 1:**
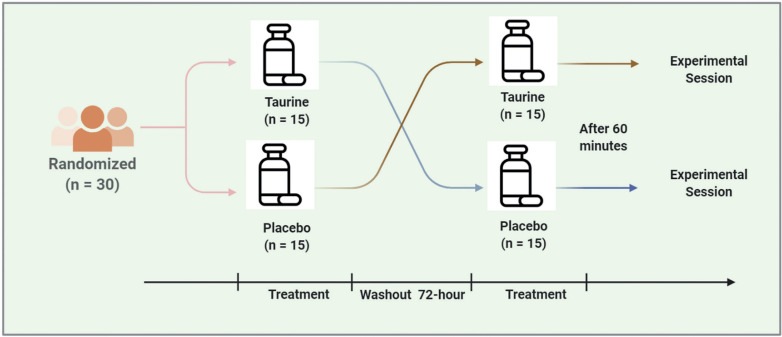
Summary of the experimental session.

Following acute supplementation, participants complete a series of physical performance tests including a Wingate anaerobic test (WanT) and a counter movement jump test (CMJ) to assess neuro-muscular fatigue. The CMJ was performed immediately before and after the WanT and 3 min post WanT. Further, blood lactate (BLa) was assessed at baseline (rest) and post-WanT (immediately and 3.5 min after completion). An overview of the experimental protocols is shown (Figure 1).

### Supplementations and Dietary Control

Participants were instructed to visit the laboratory ~90 minutes before the start of WanT and to ingest the supplement 60 minutes before the performance tests. Participants ingested either TAU (single dose of 6 g) or an isovolumetric isocaloric PLAC (single dose of 6 g of starch supplement). The supplements were provided in liquid form, mixed with 500 mL of water. TAU and PLAC supplementation was given 60 minutes before measurements because TAU plasma concentrations peak at 1 hour [39]. Seventy-two hours before the start of each experimental condition, participants were instructed to follow nutritional guidelines, which dietitians monitored to ensure similar nutrient intake before both conditions. Furthermore, participants were instructed to avoid caffeine within seventy-two hours of the experimental sessions.

### Rating of Perceived Exertion (RPE)

The Borg Scale (6–20) is a valuable indicator of personal effort and is commonly used in exercise science, also known as RPE [40]. Rating of perceived exertion was recorded immediately after each WanT.

### Blood Lactate

Blood lactate was measured at three-time points: at rest (baseline), immediately after completion of the WanT (L-post), and 3.5 minutes during recovery (L-post-3.5). Blood samples (5 μl) were taken from the tip of the index finger of the left hand using the Lactate Scout 4 (Leipzig, Germany) analyzer and following manufacturing instructions.

### Neuromuscular Fatigue

CMJ performance was measured before and after the WanT using a pre-determined protocol that has been previously described [41]. CMJ was assessed at three different time points: baseline, immediately after the WanT (CMJ post), and 3 minutes after WanT (CMJ post-3). At each time point, participants performed two CMJ separated by a 45-s rest period using a force platform (Microgate Opto Jump, Mahopac-USA): before WanT (CMJ-pre). The mean values of peak power (PP), jump total time (TT), mean power (MP), and from the two CMJ measurements were recorded. Peak power measurement is calculated automatically from the program according to the formula below [42].

$$\begin{array}{l} \text{Peak Power }(\text{Watts})=[60.7\times \text{Jump Height }(\text{cm})]\\ \;+[45.3\times \text{Body Mass }(\text{kg})]-2055 \end{array}$$

### Wingate Anaerobic Test (WanT)

WanT was conducted according to previously published protocols. The WanT was completed on a Monark cycle ergometer (Monark Classic Ergomedic 894E, Vansbro, Sweden). The participants’ feet were fixed onto the pedals and seat height was adjusted to ensure near full extension at the bottom of each pedal revolution. The seat height and position were recorded and replicated for each condition. The load on the front basket was set at 7.5% of the participant’s body mass (kg). The participants were asked to go as fast as possible before engaging the resistance. The participants then maintained an “all-out” effort for 30-s [8, 9]. Each participant was encouraged with standardized verbal encouragement throughout the WanT. During the test, power output (W) was recorded every second. Peak power output, time to reach peak power, minimum power output and average power output were extracted for data analysis. The lowest power recorded in the final 10-s of the test was defined as the minimum power. Average power was calculated separately for five-second splits, 6 times for a total of 5-s, WanT (W_split_0–5_, W_split_5–10_, W_split_10–15_, W_split_15–20_, W_split_20–25_, and W_split_25–30_).

### Familiarization

**FIG. 2:**
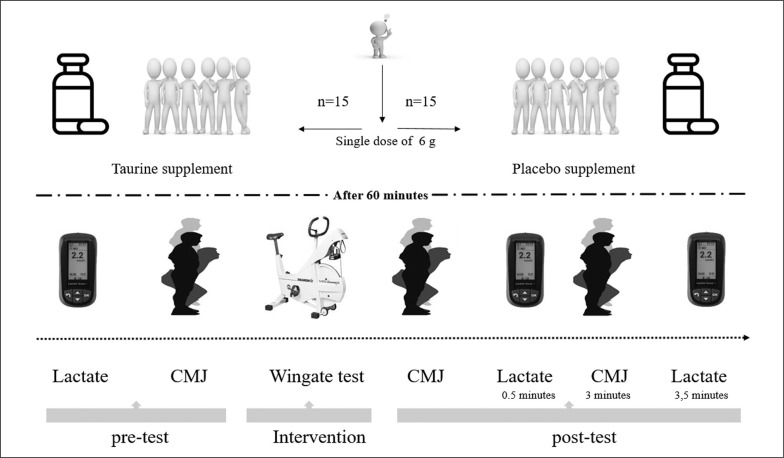
Visualization flow of the experimental procedure.

All participants participating in the research were determined within the scope of the project. Participants were already familiar with the Want test protocol as they were elite athletes. However, in order to avoid any problems, all participants were informed about the practice/ trial process and the exercise to be applied. Then, one week before the exercise protocol, all participants were brought together and the Want test protocol was applied. Thus, it has gone through the habituation/trial process. A predetermined visualized experiment flow chart was created for this study within the scope of the project. All experimental procedures (other details of the implementation/testing phase) are summarized (Figure 2).

### Statistical analysis

The data are shown as [95% CI], mean and standard deviation (SD). The significance level for all analyses was accepted as p < 0.05. The Shapiro-Wilk test was preferred because the sample sizes (n < 50) were low [43]. The WanT was analyzed using a dependent t-test following confirmation of normality via the Shapiro-Wilk test and if data were non-normally distributed, the Wilcoxon test was applied. For BLa and NMF, a supplementation by time analysis of variance for repeated measurements (ANOVA-RM) was used, whereas for split-specific mean power, a supplementation by split ANOVA-RM was used. Compliance with the sphericity assumption was checked with Mauchly’s test. In the conditions where the sphericity assumption was not met (p < 0.05), Epsilon (ε) values were examined for the degrees of freedom, Greenhouse-Geisser correction was applied for ε < 0.75, and Huyn-Feldt correction was applied for ε > 0.75. The effect size was calculated with the partial eta squared coefficient (η_p_^2^) and classified as 0.099 = small, 0.0588 = moderate, 0.1379 = large effect [44]. The effect size of Cohen’s d was calculated, with values > 0.8, 0.5–0.8, 0.2–0.5, and < 0.2 being considered high, moderate, small, and trivial, respectively for pairwise comparisons [45]. SPSS software (IBM Corp. Released 2016. IBM SPSS Statistics for Windows, Version 25.0. Armonk, NY: IBM Corp) was used for all statistical analyses.

## RESULTS

### Wingate test performance

Enhanced P_peak_ (W) (D% = 13.41; t = 4.524; p < 0.001; d = 1.71), P_peak_ (W/kg) (D% = 18.08; t = 3.534; p < 0.003; d = 1.36), P_mean_ (D% = 3.95; t = 3.787; p < 0.002; d = 1.04), P_min_ (D% = 7.89; t = 2.235; p < 0.034; d = 0.48) and along with decreased T_P_peak_ (D% = -22.93; t = 5.920; p < 0.001; d = 2.23), and RPE (D% = -10.98; t = 3.985; p < 0.002; d = 0.46) were indicated in the WanT for the TAU condition, compared to PLAC (Table 2). Consequently, there was a higher mean power output for TAU, compared to PLAC (683.90 ± 105.20 vs. 657.09 ± 119.25 W).

**TABLE 2:** Effects of TAU supplementation on WanT power output

	PLAC		TAU					

	mean ± SD	[95% CI]	mean ± SD	[95% CI]	D%	t	*p*	d
P_peak_ (W)	894.03 ± 105.47	[856.43, 931.57]	1014.7 ± 183.02	[948.51, 1079.4]	13.41	4.524	**< 0.001**	1.71
P_peak_ (W/kg)	10.84 ± 0.89	[10.32, 11.36]	12.80 ± 1.87	[11.72, 13.89]	18.08	3.534	**0.003**	1.36
P_mean_ (W)	657.09 ± 119.25	[614.42, 699.58]	683.90 ± 105.20	[645.43, 720.57]	3.95	3.787	**0.002**	1.04
P_min_ (W)	423.87 ± 93.83	[389.72, 456.28]	457.32 ± 104.85	[419.78, 494.22]	7.89	2.235	**0.034**	0.48
T_P_peak_ (s)	8.85 ± 0.77	[8.41, 9.30]	6.82 ± 1.03	[6.22, 7.41]	-22.93	5.920	**< 0.001**	2.23
RPE (score)	17.11 ± 3.77	[15.93, 18.07]	15.23 ± 4.32	[13.45, 16.55]	-10.98	3.985	**0.002**	0.46

Abbreviations: WanT: Wingate anaerobic test, TAU: taurine, PLAC: placebo, D%: percentage difference from PLAC, P_peak_: peak power, P_mean_: mean power; P_min_: minimum power, 95% CI: 95% confidence interval, T_P_peak_: time to reach, *d*: Cohen’s *d* effect size, RPE: Rating of Perceived Exertion, mean ± SD: mean ± standard deviation.

**FIG. 3:**
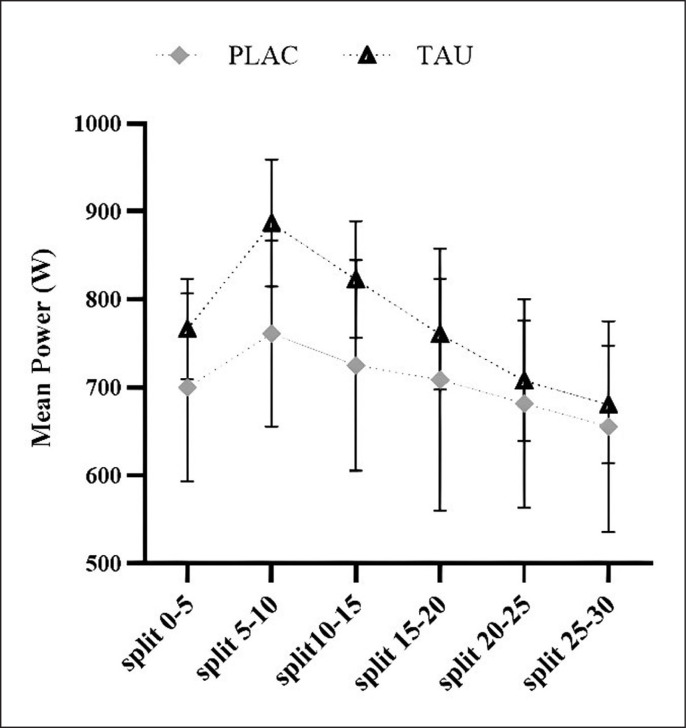
5-s split graphical representation of average power output during Wingate anaerobic test.

The findings of comparing mean power statistics across splits are detailed (Table 3 and Figure 3). An effect of supplementation (F_1,13_ = 30.65; p < 0.001; η_p_^2^ = 0.78) and Time (F_3.632, 47.21_ = 4.839; p < 0.003; η_p_^2^ =0.085), with no Supplementation × Time interaction (F_3.478, 45.21_ = 1.005; p = 0.407; η_p_^2^ = 0.24), as during WanT protocol, it was discovered for mean power measured in 6 splits of 5-s each.

**TABLE 3:** During WanT, mean power output was measured in 5-s splits.

	PLAC		TAU		Statistical significance		

	mean ± SD	[95% CI]	mean ± SD	[95% CI]	supplement	time	supplement × time
split_0–5_	700.38 ± 106.68	[662.07, 737.93]	766.59 ± 109.28	[726.99, 805.01]	**< 0.001**	**0.003**	0.407
split_5–10_^Ϯ^	761.28 ± 119.45	[718.42, 803.60]	886.67 ± 125.00	[841.27, 930.73]			
split_10–15_^Ϯ℥^	725.16 ± 109.45	[685.99, 764.20]	822.88 ± 115.21	[780.85, 863.15]			
split_15–20_^℥^	709.16 ± 148.98	[656.04, 761.96]	760.71 ± 109.25	[720.99, 799.01]			
split_20–25_^℥⅀^	681.97 ± 118.31	[638.77, 723.23]	707.85 ± 118.77	[664.77, 749.23]			
split_25–30_^Ϯ℥⅀ֆ^	655.78 ± 119.86	[614.42, 697.58]	680.71 ± 105.63	[642.43, 717.57]			

Abbreviations: WanT: Wingate anaerobic test, 95% CI: 95% confidence interval, TAU: taurine, PLAC: placebo

Ϯ : significantly different (*p* < 0.001) from split_0–5_

℥ : significantly different (*p* < 0.001) from split_5–10,_

⅀ : significantly different (*p* < 0.001) from split_15–20_

ֆ : significantly different (*p* < 0.001) from split_20–25_, mean ± SD: mean ± standard deviation.

### Blood lactate concentration

**FIG. 4:**
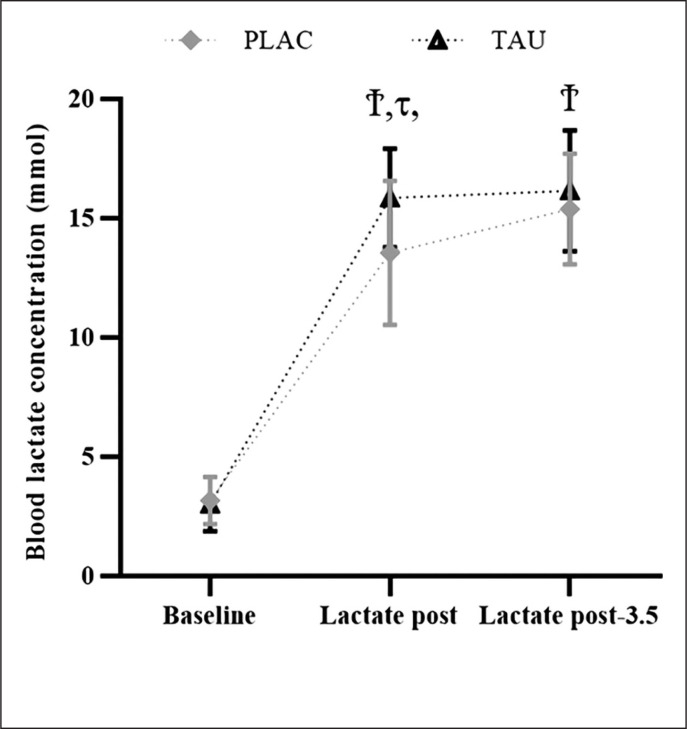
This figure shows the mean ± [95% CI] for blood lactate concentration registered at Lactate pre, Lactate post, and Lactate post-3.5 τ: significant differences between TAU and PLAC (p < 0.05), Ϯ: significant differences from Lactate pre (p < 0.05).

This study analysis unveiled an effect of Time (F_2,78_ = 301.4; p < 0.001; η_p_^2^ = 0.365), Supplementation (F_1,78_ = 4.274; p < 0.001; η_p_^2^ = 0.95), and a Supplementation × Time interaction (F_2,78_ = 2.362; p < 0.024; η_p_^2^ = 0.29). BLa was ascertained to be raised (p < 0.001) at L-post and L-post-3.5, under both the TAU and PLAC conditions. BLa was also higher in the TAU condition at L-post (∆% = 16.99; p < 0.026) and L-post-3.5 (∆% = 4.96; p < 0.041), compared to PLAC (Figure 4).

### Neuromuscular fatigue

A time effect was found for CMJ TT (F_1,96_ = 2.920; p < 0.034; η_p_^2^ = 0.78), with greater CMJ post measurement noted compared to CMJ post-3 (0.95 ± 0.21 vs. 0.69 ± 0.14; p < 0.044). The supplementation effect (F_1,06_ = 0.184; p = 0.652; η_p_^2^ = 0.09) and Supplementation × Time interaction (F_1,96_ = 0.179; p = 0.703; η_p_^2^ = 0.04) was both found to be non-significant.

**FIG. 5:**
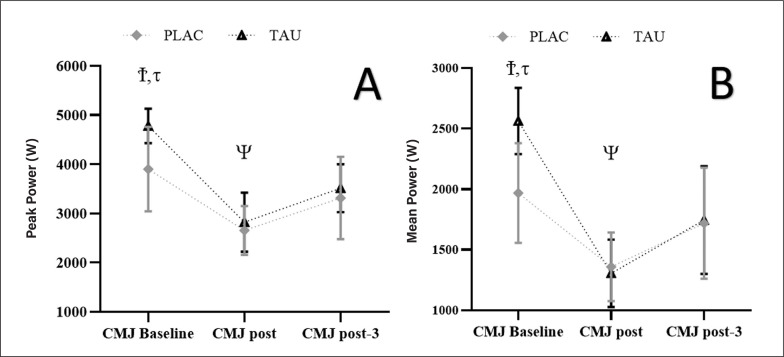
This figure shows the mean ± [95% CI] for PP (A) and MP (B) registered at CMJ pre, CMJ post, and CMJ post-3. **τ**: significant difference (p < 0.01) between TAU and PLAC, **Ψ**: significant difference (p < 0.001) compared to post-3 for TAU and PLAC, **Ϯ**: significant difference (p < 0.001) compared to post and post-3 for TAU and PLAC.

For PP, there was a Supplementation by Time interaction (F_1,96_ = 3.120; p < 0.028; η_p_^2^ = 0.34) as well as a Time effect (F_1.489, 9.547_ = 116.754; p < 0.001; η_p_^2^ = 0.92) but no Supplementation effect (F_1,06_ = 2,985; p = 0.063; η_p_^2^= 0.13). For all circumstances, greater measurements (p < 0.001) were found at CMJ pre, compared to CMJ post and CMJ post-3, while smaller values (p = 0.001) were found at CMJ post, compared to CMJ post-3. Furthermore, TAU had a greater PP at CMJ pre compared to PLAC (3.900 ± 860 vs. 4780 ± 548 W; p < 0.022; Figure 5A.

For MP, there was a Supplementation by Time interaction (F_1,96_ = 3.745; p < 0.035; η_p_^2^ = 0.26) and a Time effect F_1.863, 24.421_ = 132.754; p < 0.001; η_p_^2^ = 0.78) but no Supplementation effect (F_1,06_ = 3.234; p = 0.074; η_p_^2^ = 0.16). For all circumstances, greater values (p < 0.001) were found at CMJpre, compared to CMJ post and CMJ post-3, while smaller values (p < 0.001) were found at CMJ post, compared to CMJ post-3. Furthermore, TAU had a higher MP at CMJ pre compared to PLAC (1968 ± 411 vs. 2563 ± 247 W; p < 0.001; Figure 5B.

## DISCUSSION

The significant results from the study support our hypothesis that acute TAU supplementation enhances anaerobic power outputs without altering neuromuscular fatigue. According to our findings, TAU supplementation improves athletic performance and ameliorates athletes’ functional response to training, but it does not influence fatigue concentration.

Compared to other studies, it appears that athletes ingesting TAU can enhance WanT performance leading to higher BLa compared to placebo. For example, the results of the study conducted by Y Matsuzaki, et al. [46] support our findings. Although speculation, the higher BLa concentration following anaerobic exercise with TAU compared to PLAC may be associated with factors related to the inhibitory mechanisms of adenosine receptors causing an increase in the mean power, as well as the assumption that there may be an increase in lactate production with the increase in the need for ATP by the phosphagen and glycolytic systems. When comparing BLa concentrations, the high BLa following TAU in L-post and L-post-3.5 is evidence of increased anaerobic glycolysis [47]. Further, the PP and MP values, which we determined as anaerobic power outputs, between TAU and PLAC conditions, were not significantly different between CMJpost and CMJpost-3 time points, and it was found to be at a lower level than CMJpre in both conditions. Again, this data is supported by previous literature [48, 49]. However, it is unclear which mechanisms are involved in TAU supplementation to influence CMJ performance. When evaluating the CMJpre state, it is assumed that PCr and RPE have little impact on performance. However, ADP and H ^+^ accumulation during CMJ assessment following WanT may alter muscular contraction force. The low or fully depleted PCr concentration after WanT may partially explain the reduced performance reported in the CMJpost and CMJpost-3. Additionally, considering the chemical process of PCr resynthesis, the upward tendency to jump performance observed in CMJpost-3 is likely the result of partial resynthesis of PCr reserves [50, 51].

When the literature was examined, studies evaluating anaerobic capacity and applied in different dosages were found. According to these studies, 1 g of taurine supplementation taken 1 hour before exercise in female athletes [52] and an average of 4.3 g of taurine supplementation in elite level athletes [53] determined Wingate anaerobic capacity measurements (peak and mean power) has been found to increase. It was determined that an average of 7.5 g of taurine [54] taken 1 hour before high-intensity exercise for 1 week in healthy individuals and 1 g of taurine supplementation taken acutely in professional athletes [55], reduced blood lactate level and neuromuscular fatigue, on the contrary, 3 g of taurine supplementation daily for 8 weeks was determined to increase blood lactate level in elite swimmers [56]. Unlike these, it has been stated that 3 g of taurine [57] given to elite athletes for 10 days has no effect on blood lactate level and countermovement jump height. Since the results obtained are different from each other, more studies are needed to understand anaerobic effects more clearly.

Notably, following TAU supplementation, WanT was performed at higher power output and lower fatigue despite no effect on CMJ. TAU has positively affected intramuscular and intermuscular coordination, resulting in improved jumping performance. As the number of studies evaluating enhanced Ca^2 +^ bioavailability in myoplasm increases, and when considered with experimental studies, this may further explain these findings. Further, the participation of advanced motor units is known to serve as a synergistic process that improves jumping ability and may be altered by TAU [4, 58]. In support of these potential mechanisms, it was observed that P_peak_ and P_mean_ increased and T_P_peak_ decreased during WanT measurements in participants ingesting TAU supplementation.

WanT measurement, which lasted for 30-s, was evaluated separately in 6 splits of 5-s. P_peak_ and maximum average power were reached in the 2^nd^ split. Phosphocreatine is limited in muscle but can regenerate ATP by creatine kinase rapidly and acts as a temporary energy buffer during high-intensity exercise [51, 59]. Further, the metabolic changes (accumulation of H ^+^, pH decrease, acidosis) that occur with the contraction of the muscles at maximum capacity during WanT affect muscle contraction function and cause fatigue and decreased power output production in the last seconds of the second split of WanT [60]. Further, TAU supplementation improves calcium permeability in sarcoplasmic reticulum of type I and type II muscle fibers, improves contractile function by increasing the sensitivity of myofilaments to calcium, and thus can increase power output [58, 61]. Additionally, according to an *in vivo* study, TAU supplementation assists with Ca^2 +^ collection in the sarcoplasmic reticulum, likely including a rise in SR Ca^2 +^ pump function or the Sarco/endoplasmic reticulum Ca-ATPase affinity for Ca^2 +^ in human skeletal muscle fibers [58]. In line with these theories, TAU positively enhanced performance. However, when evaluated in terms of its effect on performance, it is unclear whether the mechanisms altered the inter or intramuscular environments [4, 58, 62].

Furthermore, TAU consumption did not alter neuromuscular fatigue; PP and MP were obtained due to CMJ measurement, peak, mean, minimum power (P_peak_, P_mean_, and P_min_), and time to peak power (T_P_peak_). Currently, limited comparison data is available regarding TAU on neuromuscular fatigue following anaerobic exercise [49, 52, 63]. According to R Warnock, et al. [49], 50 mg /kg TAU ingestion increased mean peak power (MPP), peak power (PP), and mean power (MP) outputs compared to the placebo as a result of the WanT protocol. Similarly, in another study examining the effect of taurine doses of 2, 4, and 6 g on anaerobic performance, it was reported that an increase was observed in the mean and peak power outputs measured as a result of WanT performance with 6 g TAU ingestion [63]. In contrast, 1 g TAU ingestion did not significantly increase WanT performance outputs (such as peak and mean power) compared to placebo [52].In these studies, the effects of caffeine and taurine supplementation were also examined which may have influenced the findings [64, 65].

Studies have shown that TAU affects Ca^2 +^ homeostasis and K_ATP_ channel activity in different cell types, anti-oxidant properties by inhibiting ROS in mitochondria, osmoregulation, anti-inflammatory effects, and regulates glucose homeostasis. TAU is found in higher concentrations in pancreatic islets and regulating insulin secretion in response to food by increasing Ca^2 +^ mobilization in pancreatic β-cells. Considering the Ca^2 +^ and K_ATP_ regulatory effect of taurine, cell ATP production is initiated by stimulus-secretory coupling and glucose entry via glucose transporters (GLUT)-2 in β-cells. This is followed by the complete sugar metabolism and ATP production. An increase in the ATP/ADP ratio leads to the closure of ATP-sensitive potassium (K_ATP_) channels, depolarization of the plasma membrane, and opening of voltage-sensitive Ca^2 +^ channels. Ca^2 +^ influx activates the exocytotic machinery that coordinates insulin granule migration and fusion [19, 66].

Studies show that taurine affects increasing endurance exercise performance. For example, taurine aids in sarcoplasmic reticulum Ca^2 +^ transport after intramuscular entry, with muscle performance attributed to taurine-facilitated Ca^2 +^ transport by cardiac and skeletal myocytes [67]. In another study, it was seen that taurine exerts a stabilizing effect by acting on the mitochondrial matrix. Thanks to this stabilizing effect, it increases the efficiency of the ATP cycle in the muscle cell [68]. Based on this result, taurine has also been shown to play a role as an anti-oxidative. Indeed, inhibition of taurine uptake in taurine transporter knockout mice significantly shortened extinction time [69].

Although taurine is known to be abundant in skeletal muscle, its specific function is currently poorly understood [70]. TAU is thought to play a role in aerobic exercise and mitochondrial function, as higher concentrations in skeletal muscle have been reported in trained (~64 mmol kg^−1^ dw) and untrained (~50 mmol kg^−1^ dw) participants [71]. However, our experimental results suggest that TAU supplementation may improve anaerobic performance. Considering the regulatory role of taurine in all these pathways, it is thought that the energy deficit, especially in athletes, can be eliminated very quickly by ATP production after taurine supplementation this way.

## CONCLUSIONS

In conclusion, acute TAU supplementation augments anaerobic performance in elite speed skaters but does not alter neuromuscular recovery.

### Practical Applications

The nature of short-track skating has a crouching position and due to this position, when enough oxygen does not reach the muscles, fatigue occurs. In addition, the power generation of the lower extremity in this sport is also a factor that contributes to success.

Considering that the buffering effect of taurine supplement reduces lactate level and supports power production of muscle myofibrils by increasing calcium release from the sarcoplasmic reticulum, it can be said that this supplement supports anaerobic capacity and its use can be recommended to short-track skaters.

More studies are needed to examine the effect of taurine on anaerobic sports branch performance. Increasing the number of studies; is thought that will shed light on the usability of taurine supplementation to athletes, conditioners, and trainers in the future.

### Limitation and Further Research

While looking at the acute effect of taurine supplementation is the limitation of this study, its strength is to conducted with well-trained athletes. In future studies, aerobic and anaerobic effects can be examined in different sports branches in chronic use and at different doses (safe and tolerable) and studies can be expanded by comparing these effects. It is seen that there is a deficiency in the literature in terms of studies on well-trained athletes. Starting from this point, it is thought that experimental studies on taurine should focus on well-trained athletes.

### Ethical approval

All protocols and procedures were carried out in according to the Declaration of Helsinki and were approved by the Atatürk University Ethics Committee (E-70400699–050.02.04–2100316289, 2021/10).

### Informed consent

Informed consent was obtained from all individual participants included in the study.
